# The Inhibition of CD39 and CD73 Cell Surface Ectonucleotidases by Small Molecular Inhibitors Enhances the Mobilization of Bone Marrow Residing Stem Cells by Decreasing the Extracellular Level of Adenosine

**DOI:** 10.1007/s12015-019-09918-y

**Published:** 2019-09-13

**Authors:** Mateusz Adamiak, Kamila Bujko, Katarzyna Brzezniakiewicz-Janus, Magda Kucia, Janina Ratajczak, Mariusz Z. Ratajczak

**Affiliations:** 1 Stem Cell Institute at James Graham Brown Cancer Center, University of Louisville, 500 S. Floyd Street, Rm. 107, Louisville, KY 40202 USA; 2 Center for Preclinical Studies and Technology, Department of Regenerative, Medicine Warsaw Medical University, Warsaw, Poland; 3 Department of Hematology, University of Zielona Gora, Hospital Gorzow Wlkp, Zielona Góra, Poland

**Keywords:** Sterile inflammation, Extracellular nucleotides, CD39, CD73, Complement cascade, ATP, Adenosine, Stem cell mobilization, MSCs, EPCs, VSELs

## Abstract

We have recently demonstrated that purinergic signaling in bone marrow (BM) microenvironment regulates mobilization of hematopoietic stem progenitor cells (HSPCs), mesenchymal stroma cells (MSCs), endothelial progenitor cells (EPCs), and very small embryonic like stem cells (VSELs) into the peripheral blood (PB). While extracellular adenosine triphosphate (ATP) promotes mobilization, its metabolite extracellular adenosine has an opposite effect. Since ATP is processed in extracellular space to adenosine by ectonucleotidases including cell surface expressed CD39 and CD73, we asked if inhibition of these enzymes by employing in vivo small molecular inhibitors ARL67156 and AMPCP of CD39 and CD73 respectively, alone or combined could enhance granulocyte stimulating factor (G-CSF)- and AMD3100-induced pharmacological mobilization of stem cells. Herein we report that pre-treatment of donor mice with CD39 and CD73 inhibitors facilitates the mobilization of HSPCs as well as other types of BM-residing stem cells. This data on one hand supports the role of purinergic signaling in stem cell trafficking, and on the other since both compounds are not toxic against human cells, they could be potentially employed in the clinic to enhance the mobilization of BM residing stem cells for clinical purposes.

**Electronic supplementary material:**

The online version of this article (10.1007/s12015-019-09918-y) contains supplementary material, which is available to authorized users.

## Introduction

Mechanisms that govern the egress of cells from bone marrow (BM) into peripheral blood (PB) are still not very well characterized and several overlapping redundant mechanisms have been proposed [1–7]. Our group postulates that this process is initiated mainly by the activation of the cellular and humoral arm of innate immunity in the BM microenvironment in response to pro-mobilizing stimuli [2, 5, 8]. An important role in triggering this process plays adenosine triphosphate (ATP), secreted from activation by pro-mobilizing drugs cells that trigger via Nlrp3 inflammasome activation of complement cascade (ComC) in the BM microenvironment [9–11]. In fact, as demonstrated, ComC cleavage fragments such as C5a, _desArg_C5a and C5b-C9 are important mediators regulating egress of hematopoietic stem/progenitors cells (HSPCs) [2, 8, 12] as well as other BM-residing stem/progenitor cells including mesenchymal stroma cells (MSCs) [13], endothelial progenitors (EPCs) [14, 15], and rare very small embryonic like stem cells (VSELs) into PB [16, 17].

The identification of extracellular ATP as an important initiator of stem cell mobilization established a role of purinergic signaling in this process. In the extracellular microenvironment, ATP is metabolized to adenosine that as reported has immunosuppressive properties and what is relevant for current work is an inhibitor of stem cells mobilization [9–11]. ATP degradation to adenosine is mediated by ectonucleotidases and two of them cell surface expressed CD39 (Ectonucleoside triphosphate diphosphohydrolase-1 or NTPDase1) and CD73 (5′-nucleotidase (5’-NT), also known as ecto-5′-nucleotidase) play a crucial role [18, 19]. Both these ectonucleotidases are expressed by cells in the BM microenvironment [18, 19].

In our previous work, we have demonstrated that CD73-KO mice are easy mobilizers of stem cells in response to granulocyte colony stimulating factor (G-CSF) and CXCR4 receptor antagonist AMD3100, what has been explained by a decrease in extracellular availability of adenosine [9, 10]. To support this further, injection of mice with biologically tolerable bolus of adenosine had a negative effect on stem cell mobilization and upregulated expression of heme oxygenase-1 (HO-1) in BM cells, a known inhibitor of ComC activation and mobilization of HSPCs [20]. HO-1 has been also reported as an important inhibitor of Nlrp3 inflammasome that as we recently proposed orchestrates via innate immunity cells the ATP-mediated mobilization process [11].

Since there are available and tested in in vivo small molecular inhibitors of CD39 and CD73, ARL67156 and AMPCP, respectively [19, 20]; we become interested if these compounds by lowering extracellular level of adenosine could be employed to enhance egress of HSPCs and other stem cells from BM into PB.

We demonstrate for a first time that inhibition of CD39 and CD73 enhances mobilization of HSPCs as well as MSCs, EPCs, and VSELs. Moreover, since we found expression of CD39 and CD73 on the surface of HSPCs, and adenosine P1 receptors are also expressed by these cells, further studies are needed to evaluate potential autocrine, paracrine role of adenosine in this process.

## Material and Methods

### Animals

Pathogen-free, 4–6-week-old C57BL/6 J mice were purchased from the Central Laboratory for Experimental Animals, Medical University of Warsaw or the Jackson Laboratory (Bar Harbor, ME, USA) at least 2 weeks prior to experiments. Animal studies were approved by the Animal Care and Use Committee of the Warsaw Medical University (Warsaw, Poland) and University of Louisville (Louisville, KY, USA).

### CD39 and CD73 Inhibitors

Inhibitors of CD39 - ARL67156–6-N,N-Diethyl-β-γ-dibromomethylene-D-adenosine-5′-triphosphate trisodium salt hydrate and CD73 - AMPCP - Adenosine 5′-(α,β-methylene)diphosphate sodium salt were purchased from Tocris (Bristol, UK) and dissolved as recommended by producer.

### In Vivo Mobilization Studies

Mice were injected once by CD39 (2 mg/kg, intraperitoneal injection (IP) or/and CD73 inhibitors (4 mg/kg i.p.) 3 h before termination of mobilization with G-CSF (Amgen, Thousand Oaks, CA, USA) for 3 days at 100 μg/kg per day by subcutaneous injection (SC) or with AMD3100 (Sigma-Aldrich, St. Louis, MO, USA) for 1 day at 5 mg/kg IP. At 6 h after the last G-CSF injection, 1 h after AMD3100 injection, the mice were bled from the retro-orbital plexus for plasma and hematology analysis, and PB was obtained from the vena cava (with a 25-gauge needle and 1-ml syringe containing 250 U heparin). MNCs were obtained by hypotonic lysis of RBCs in BD Pharm Lyse buffer (BD Biosciences) as described [10–12].

### Detection of CD39 and CD73 by FACS

Murine cells were isolated from pathogen-free C57BL/6 mice, suspended in BD Pharm Lyse buffer (BD Biosciences, San Jose, CA, USA) to remove RBCs, and washed and resuspended in RPMI medium. The CD39 and CD73 expression was evaluated on Sca-1^+^c-Kit^+^ Lin^−^ (SKL) BM cells after incubation with primary mouse monoclonal anti-CD39 (PE-Cy7, clone 24DMS1; Invitrogen, Carlsbad, CA, USA) and anti-CD73 (APC, clone TY/11.8; BioLegend, San Diego, CA, USA) antibodies.

### Evaluation of HSPC Mobilization

The following strategies were employed to evaluate mobilization efficacy:

- *PB parameter counts*. To obtain white and red blood cell counts, 50 μl of PB was taken from the retro-orbital plexus of mice into microvette EDTA-coated tubes (Sarstedt Inc., Newton, NC, USA) and run on a HemaVet 950FS hematology analyzer (Drew Scientific Inc., Oxford, CT, USA) within 1 h of collection as described [10–12].
- *Fluorescence-activated cell sorting (FACS) analysis of circulating stem cells.* For staining of Sca-1^+^/c-Kit^+^Lin^−^/ (SKL cells), Lin^−^/CD45^−^/CD31^−^/CD90^+^ (MSCs), Lin^−^/CD45^−^/CD31^+^ (EPCs), and Sca-1^+^/Lin^−^/CD45^−^ (VSELs) the following monoclonal antibodies were used: FITC–anti-CD117 (also known as c-Kit, clone 2B8; BioLegend, San Diego, CA, USA) and PE–Cy5–anti-mouse Ly-6 A/E (also known as Sca-1, clone D7; eBioscience, San Diego, CA, USA). All anti-mouse lineage marker antibodies, including anti-CD45R (also known as B220, clone RA3-6B2), anti-Ter-119 (clone TER-119), anti-CD11b (clone M1/70), anti-T cell receptor β (clone H57–597), anti-Gr-1 (clone RB6-8C5), anti-TCRγδ (clone GL3), and anti-CD45 (clone 30-F11), conjugated with PE; anti-CD31 (clone MEC 13.3), conjugated with APC; and anti-CD90.2 (clone 30-H12), conjugated with BV510, were purchased from BD Biosciences. Staining was performed in RPMI-1640 medium containing 2% FBS. All monoclonal antibodies were added at saturating concentrations, and the cells were incubated for 30 min on ice, washed twice, and analyzed with an LSR II flow cytometer (BD Biosciences) as described [10–12].
- *Evaluation of mobilized clonogenic progenitor cells.* For evaluation of circulating colony-forming granulocyte/macrophage (CFU-GM) and SKL cells, the following formulas were used: (number of white blood cells [WBCs] × number of CFU-GM colonies)/number of WBCs plated = number of CFU-GM per ml of PB; and (number of WBCs × number of SKL cells)/number of gated WBCs = number of SKL cells per μl of PB as described [10–12].

### Fibronectin Cell-Adhesion Assay

Murine BMMNCs pre-treated with adenosine for 1 h were resuspended in RPMI 1640 plus 0.5% bovine serum albumin (BSA) medium (5 × 10^4^cells/100 μl). Cell suspensions were added directly to 96-well plates coated before the experiment with fibronectin (10 μg/ml), incubated overnight at 4 °C, and then blocked with medium containing 0.5% BSA for 2 h at 37 °C. Non-adherent cells were then washed from the wells, and all adherent cells were counted using an inverted microscope [10–12].

### Transwell Migration Assay

WT mice BMMNCs preincubated with adenosine or PBS (control) were resuspended in assay medium (RPMI-1640 with 0.5% BSA). Assay medium (650 μl), alone or containing stromal-derived growth factor 1 (SDF-1, 10 ng/ml), sphingosine-1-phosphate (S1P, 0.1 μM), ceramide-1-phosphate (C1P, 100 μM), or adenosine triphosphate (ATP, 0.25 μg/ml) was added to the lower chambers of a Costar Transwell 24-well plate (Corning Costar, Cambridge, MA, USA). Aliquots of cell suspension (1 × 10^6^ cells per 100 μl) were loaded onto the upper chambers with 5-μm pore filters and then incubated for 3 h (37 °C, 5% CO_2_). Aliquots of BMMNCs from the lower chambers were harvested and scored by FACS analysis. Briefly, the cells were gated according to their forward-scatter (FSC) and side-scatter (SSC) parameters and counted during a 30-s acquisition at a high flow rate. The rest of the BMMNCs recovered from the lower chamber were resuspended in human methylcellulose base medium provided by the manufacturer (R&D Systems), supplemented with murine GM-CSF (25 ng/ml) and IL-3 (10 ng/ml) for determining the number of CFU-GM colonies. Cultures were incubated for 7 days (37 °C, 95% humidity, and 5% CO_2_), at which time they were scored under an inverted microscope for the number of colonies [10–12].

### Statistical Analysis

All results are presented as mean ± SD. Statistical analysis of the data was done using Student’s t test for unpaired samples (Excel, Microsoft Corp., Redmond, WA, USA) with a value of *p* ≤ 0.05 considered significant.

## Results

### CD39 and CD73 Are Expressed on Murine HSPCs Cells

**Fig. 1:**
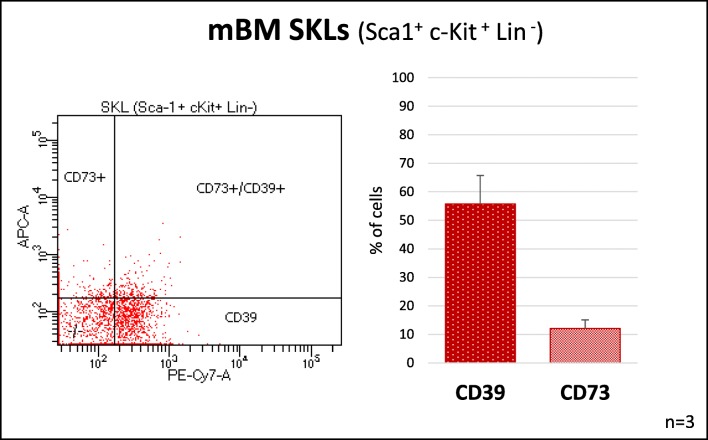
Expression of CD39 and CD73 on murine HSPCs. To analyze expression of CD39 and CD73 BMMNCs were isolated from WT mice and stained with antibodies detecting SKL cells (Sca-1^+^/c-Kit^+^/Lin^−^) and additionally with anti-CD39 and anti-CD73 antibodies. Figure shows representative dot plot and graph with pooled results from three independent stainings

CD39 and CD73 ectonucleotidases that metabolize extracellular ATP to adenosine are expressed on several types of cells in the BM microenvironment. Herein, we become interested in the expression of these enzymes on the surface of murine Sca-1^+^ Lin^−^ c-Kit^+^ (SKL) cells that are enriched for HSPCs. We noticed that both enzymes are expressed on these cells, however a level of CD39 that processes the degradation of ATP to AMP was higher than CD73 that is involved in the generation from AMP of final ATP metabolite adenosine (Fig. 1). Based on fact that CD39 and CD73 proteins are expressed on BM cells including HSPCs we employed small molecular inhibitors of both ectonucleotidases.

### Inhibition of CD39 and CD73 Ectonucleotidases by Small Molecular Inhibitors Enhances Mobilization of HSPCs

**Fig. 2:**
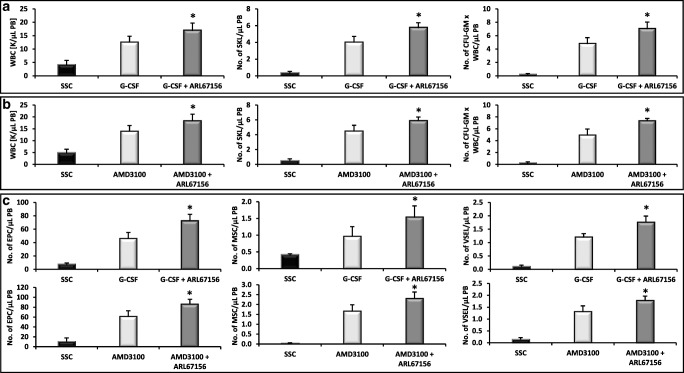
Impact of CD39 on murine HSPCs mobilization. For mobilization studies, mononuclear cells were isolated from WT mice treated with ARL67156 after 6 h following 3 days of G-CSF mobilization (**Panels A, C**) or 1 h after 1 dose of AMD3100 mobilization (**Panels B, C**), control mice received inhibitor vehicle. **Panels A, B**. The numbers of WBCs, SKL (Sca-1^+^/c-kit^+^/Lin^−^) cells, and CFU-GM clonogenic progenitors were evaluated in PB. **Panel C**. The numbers of MSCs (Lin^−^/CD45^−^/CD31^−^/CD90^+^), EPCs (Lin^−^/CD45^−^/CD31^+^), and VSELs (Sca-1^+^/Lin^−^/CD45^−^) in PB. WT (SSC) represents mice under steady-state conditions. Results from two independent experiments (*n* = 6 animals per each group) are pooled together. *p < 0.05; comparing mobilized WT with mobilized WT administered with ARL67156

**Fig. 3:**
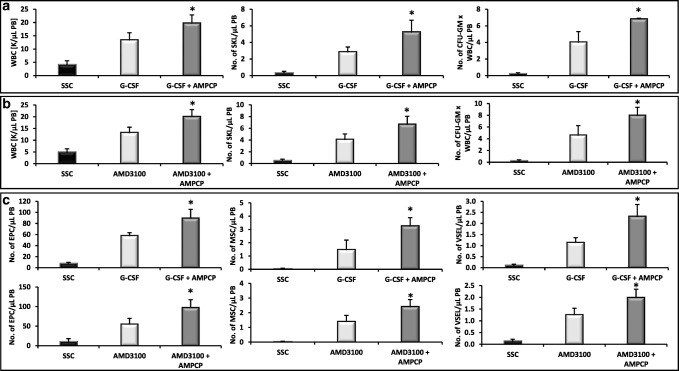
Impact of CD73 on murine HSPCs mobilization. For mobilization studies, mononuclear cells were isolated from WT mice treated with AMPCP after 6 h following 3 days of G-CSF mobilization (**Panels A, C**) or 1 h after 1 dose of AMD3100 mobilization (**Panels B, C**), control mice received inhibitor vehicle. **Panels A, B**. The numbers of WBCs, SKL (Sca-1^+^/c-kit^+^/Lin^−^) cells, and CFU-GM clonogenic progenitors were evaluated in PB. **Panel C**. The numbers of MSCs (Lin^−^/CD45^−^/CD31^−^/CD90^+^), EPCs (Lin^−^/CD45^−^/CD31^+^), and VSELs (Sca-1^+^/Lin^−^/CD45^−^) in PB. WT (SSC) represents mice under steady-state conditions. Results from two independent experiments (n = 6 animals per each group) are pooled together. *p < 0.05; comparing mobilized WT with mobilized WT administered with AMPCP

Prior to mobilization studies, mice were injected with non-toxic doses of CD39 and CD73 inhibitors (Supplementary Figs. 1 and 2) as described in Materials and Methods section and subsequently mobilized by G-CSF or AMD3100. Figure 2a and 3a show mice mobilized with G-CSF and Fig. 2b and 3b with AMD3100. The number of mobilized white blood cells, HSCs, SKL cells, and CFU-GM was significantly higher in mice exposed to CD39 and CD73 inhibitors as compared to control mice. This data shows that both small molecular inhibitors are non-toxic at employed doses against HSPCs and could become potential mobilization enhancing drugs.

**Fig. 4:**
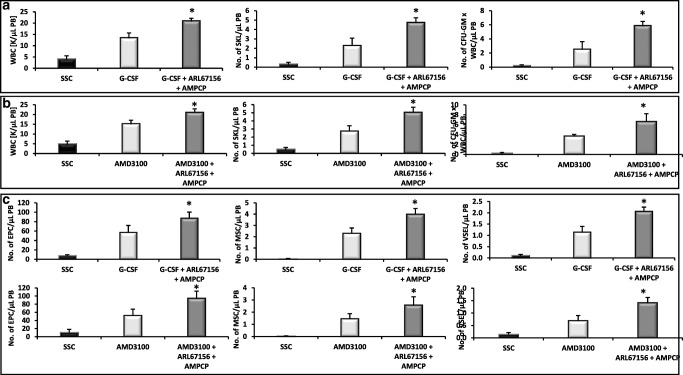
Impact of CD39 and CD73 on murine HSPCs mobilization. For mobilization studies, mononuclear cells were isolated from WT mice treated with ARL67156 and AMPCP after 6 h following 3 days of G-CSF mobilization (**Panels A, C**) or 1 h after 1 dose of AMD3100 mobilization (**Panels B, C**), control mice received inhibitor vehicle. **Panels A, B**. The numbers of WBCs, SKL (Sca-1^+^/c-kit^+^/Lin^−^) cells, and CFU-GM clonogenic progenitors were evaluated in PB. **Panel C**. The numbers of MSCs (Lin^−^/CD45^−^/CD31^−^/CD90^+^), EPCs (Lin^−^/CD45^−^/CD31^+^), and VSELs (Sca-1^+^/Lin^−^/CD45^−^) in PB. WT (SSC) represents mice under steady-state conditions. Results from two independent experiments (n = 6 animals per each group) are pooled together. *p < 0.05; comparing mobilized WT with mobilized WT administered with ARL67156 and AMPCP

Based on this encouraging data, we preconditioned mice with both inhibitors combined together and then mobilized them with G-CSF (Fig. 4a) or AMD3100 (Fig. 4b). Again, the number of mobilized white blood cells, HSCs, SKL cells, and CFU-GM was significantly higher in mice exposed to both CD39 and CD73 inhibitors as compared to control mice, but we did not observe significant improvement over application of both inhibitors separately.

### Inhibition of CD39 and CD73 Ectonucelotidases by Small Molecular Inhibitors Enhances the Mobilization of MSCs, EPCs, and VSELs

Since during the mobilization process there are released into PB also other types of stem cells, we evaluated if preconditioning of mice with CD39 and CD73 inhibitors will enhance G-CSF and AMD3100-induced mobilization of MSCs, EPCs, and VSELs. We noticed that animals exposed to ARL67156 and AMPCP mobilized in addition to HSPCs also much better other types of BM-residing stem/progenitor cells, including MSCs and EPCs as well as rare population of VSELs (Fig. 2c and Fig. 3c). A similar effect has been observed if both inhibitors were employed together (Fig. 4c). This data shows that both small molecular inhibitors at employed doses could become mobilization enhancing drugs to obtain other types of BM-residing stem cells for application in regenerative medicine.

### Adenosine Inhibits Migration of BM Cells and Increases their Adhesion

**Fig. 5:**
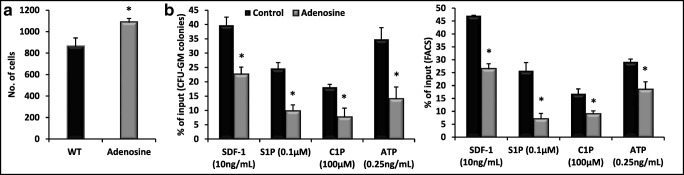
The influence of adenosine on chemotaxis and adhesion of murine BMMNCs. The chemotactic responsiveness of BMMNCs (**Panel B**) to SDF-1, S1P, C1P, and ATP gradients by clonogenic CFU-GM progenitors (left panel) and FACS (right panel). Results are combined from three independent experiments and shown as a percent of input (5% of insert). *p > 0.05. **Panel A**, the effect of adenosine on the adhesion of murine BMMNCs to fibronectin- coated plates. The results are shown as the number of adherent cells. Data from three separate experiments are pooled together. *p < 0.01

**Fig. 6:**
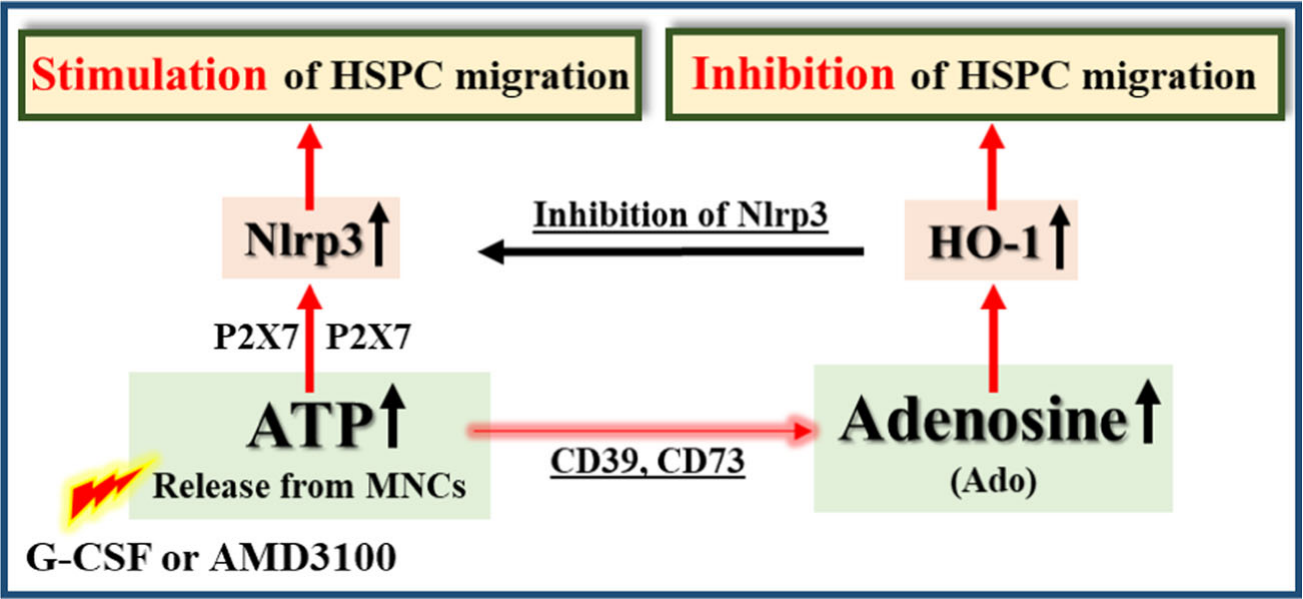
Bone marrow cells belonging to innate immunity including monocytes, granulocytes, and dendritic cells release in response to pro-mobilizing drugs (G-CSF or AMD3100) ATP into extracellular space that after binding to P2X7 receptor activates Nlrp3 inflammasome [11, 22, 23]. ATP-P2X7-Nlrp3 inflammasome axis initiates sequence of events inducing sterile inflammation in the BM microenvironment that promotes egress of stem cells into peripheral blood. At the same time, ATP is metabolized by CD39 and CD73 ectonucleotidases to adenosine that has an opposite inhibitory effect by upregulating intracellular HO-1 that inhibits stem cell migration and activation of Nlrp3 inflammasome [9, 10]

Mobilization of cells from BM into PB requires active migration to gradient of chemoattractants present in PB such as stromal derived factor-1 (SDF-1) and more important sphingosine-1 phosphate (S1P), ceramide-1 phosphate (C1P), and extracellular ATP [2]. Figure 5a shows that adenosine increases adhesion of HSPCs to fibronectin covered plates and at the same time, as shown in Fig. 5b, inhibits migration of HSPCs to gradient of SDF-1, S1P, C1P, and ATP. This data explains why adenosine promotes retention of HSPCs in BM microenvironment by increasing adhesion of these cells and attenuating their migration to gradients of HSPCs chemoattractants expressed in peripheral blood. In addition, as reported adenosine inhibits Nlrp3 inflammasome that is required for optimal mobilization of HSPCs [10, 11, 21] and Fig. 6.

## Discussion

The seminal observation of our work is that the inhibition of CD39 and CD73 ectonucleotidases enhances mobilization of HSPCs as well as other types of BM-residing stem cells including MSCs, EPCs, and VSELs. Thus, this work has some potential clinical implications.

Innate immunity as postulated plays an important role in the mobilization of BM residing cells during infections, tissue organ injury, and pharmacological mobilization [2]. Pharmacological mobilization is an important mean to harvest HSPCs for hematopoietic transplants [1–7]. One of the problems with clinical mobilization of patients as donors of HSPCs for transplantation is the fact that a significant percentage of them are poor mobilizers, and more efficient mobilization strategies are needed [1]. Therefore, in order to develop better mobilization protocols, we have to better understand the mobilization process itself at the molecular and cellular levels. Moreover, beside HSPCs also other BM-residing cells such as MSCs, EPCs, and VSELs could be harvested after mobilization from PB for potential applications in regenerative medicine approaches [2].

Egress of stem cells from BM is orchestrated by activation of purinergic signaling that involves release of adenosine triphosphate (ATP) from activated BM cells belonging to innate immunity - including granulocytes, monocytes, and as recently demonstrated also dendritic cells [2, 3]. Extracellular ATP triggers, after binding to purinergic P2X7 receptor, a sequence of events leading to the activation of Nlrp3 inflammasome [11, 21–25] that activates in the BM microenvironment complement cascade (ComC) in which cleavage products C5a, _desrArg_C5a, and C5bC9 are required for optimal egress of HSPCs from BM into PB [2, 8–12].

It has been demonstrated that while extracellular ATP promotes mobilization by activating Nlrp3 inflammasome after binding to P2X7 purinergic receptor [11], its metabolite extracellular adenosine has an opposite effect by activating intracellular heme oxygenase-1 (HO-1) in HSPCs that as we reported inhibits migration of these cells [20] and in addition also directly inhibits Nlrp3 inflammasome [21] (Fig. 6). To support this, herein we demonstrate that adenosine inhibits the migration of HSPCs to important chemoattractants present in PB involved in stem cell mobilization (S1P, C1P, SDF-1 and ATP) [2]. At the same time, it increases adhesion of HSPCs to fibronectin, that indicates its positive effect on the retention of stem cells in the BM microenvironment.

On other hand it has been reported that adenosine plays an important role in zebra fish hematopoiesis regulating number and expansion of HSPCs [26]. Despite this fact, we do not expect that one time injection of CD39 or CD73 inhibitors of extracellular adenosine synthesis would have significant side effect on a pool of HSPCs in mobilized donor. This however, requires further studies.

The degradation of ATP to adenosine in extracellular space by hydrolysis is regulated by several ectonucleotidases (E-NTPDases) [18]. In more details, the degradation of ATP to ADP, AMP, and finally adenosine is regulated by enzymatic processing by two E-NTPDases expressed on the surface of hematopoietic cells. Accordingly, ATP is first hydrolyzed stepwise to ADP, AMP by triphosphate diphosphohydrolases (E-NTPDases) known as CD39, and finally by ecto-5′-nucleotidase (eN) known as CD73 to adenosine. Both CD39 and CD73 are expressed on cells in bone marrow microenvironment and as demonstrated in this work we confirmed the presence of both ectonucleotidases on the surface of murine Sca-1^+^c-kit^+^lin^−^ cells that are enriched for HSPCs. This may support a presence of autocrine regulatory loops involving ATP and adenosine interacting with P2X and P1 receptors expressed by these cells [19].

In our studies, we employed non-toxic doses of inhibitors to block activity of these enzymes - small molecular inhibitors - AMPCP and ARL 67156, respectively, and asked if these inhibitors could be employed in vivo to facilitate pharmacological mobilization of HSPCs as well as other BM-residing stem cell populations.

Herein we report that the inhibition of CD39 or/and CD73 by small molecule inhibitors of this cell-surface-expressed enzymes facilitates egress of cells from the BM and may provide the basis for new and more efficient mobilization strategies. However, we are also aware that beside CD39 and CD73 there are also few other NTPDases involved in the metabolism of ATP such as NTPDase 3, 5, 6 and 8 [18, 19], and further studies would answer if they could be also employed as mobilization facilitating agents. Finally, in addition to CD39 and CD73 inhibitors employed in this study, there are several other similar small molecular compounds targeted against both ectonucleotidases that are worth to be tested for their potential application in stem cell mobilization.

## Electronic supplementary material

Supplementary Figure 1 Measurement of ARL67156 toxicity. Murine BMMNC and human CD34+ cells were incubated for 1 h with different doses of CD39 inhibitor, than were resuspended in human methylcellulose base medium, supplemented with GM-CSF (25 ng/ml) and IL-3 (10 ng/ml) for determining the number of CFU-GM colonies and with thrombopoietin (TPO, 100 ng/ml) and IL-3 (10 ng/ml) for burst-forming unit-erythroid (BFU-E). Cultures were incubated for 7 and 14 days respectively (37 °C, 95% humidity, and 5% CO_2_), at which time they were scored under an inverted microscope for the number of colonies. Results from three independent experiments plated in duplicates are pooled together. (PPTX 54 kb)

Supplementary Figure 2 Measurement of AMPCP toxicity. Murine BMMNC and human CD34+ cells were incubated for 1 h with different doses of CD73 inhibitor, than were resuspended in human methylcellulose base medium, supplemented with GM-CSF (25 ng/ml) and IL-3 (10 ng/ml) for determining the number of CFU-GM colonies and with thrombopoietin (TPO, 100 ng/ml) and IL-3 (10 ng/ml) for burst-forming unit-erythroid (BFU-E). Cultures were incubated for 7 and 14 days respectively (37 °C, 95% humidity, and 5% CO_2_), at which time they were scored under an inverted microscope for the number of colonies. Results from three independent experiments plated in duplicates are pooled together. (PPTX 53 kb)
