# Supplementary material for: The Inhibition of CD39 and CD73 Cell Surface Ectonucleotidases by Small Molecular Inhibitors Enhances the Mobilization of Bone Marrow Residing Stem Cells by Decreasing the Extracellular Level of Adenosine
Source: Stem Cell Rev Rep. 2019 Sep 13;15(6):892–9. doi: 10.1007/s12015-019-09918-y (PMC6925070; doi:10.1007/s12015-019-09918-y)
Supplement: Supplementary file 1 — Measurement of ARL67156 toxicity. Murine BMMNC and human CD34+ cells were incubated for 1 h with different doses of CD39 inhibitor, than were resuspended in human methylcellulose base medium, supplemented with GM-CSF (25 ng/ml) and IL-3 (10 ng/ml) for determining the number of CFU-GM colonies and with thrombopoietin (TPO, 100 ng/ml) and IL-3 (10 ng/ml) for burst-forming unit-erythroid (BFU-E). Cultures were incubated for 7 and 14 days respectively (37 °C, 95% humidity, and 5% CO2), at which time they were scored under an inverted microscope for the number of colonies. Results from three independent experiments plated in duplicates are pooled together. (PPTX 54 kb) [file 12015_2019_9918_MOESM1_ESM.pptx]

## Slide 1
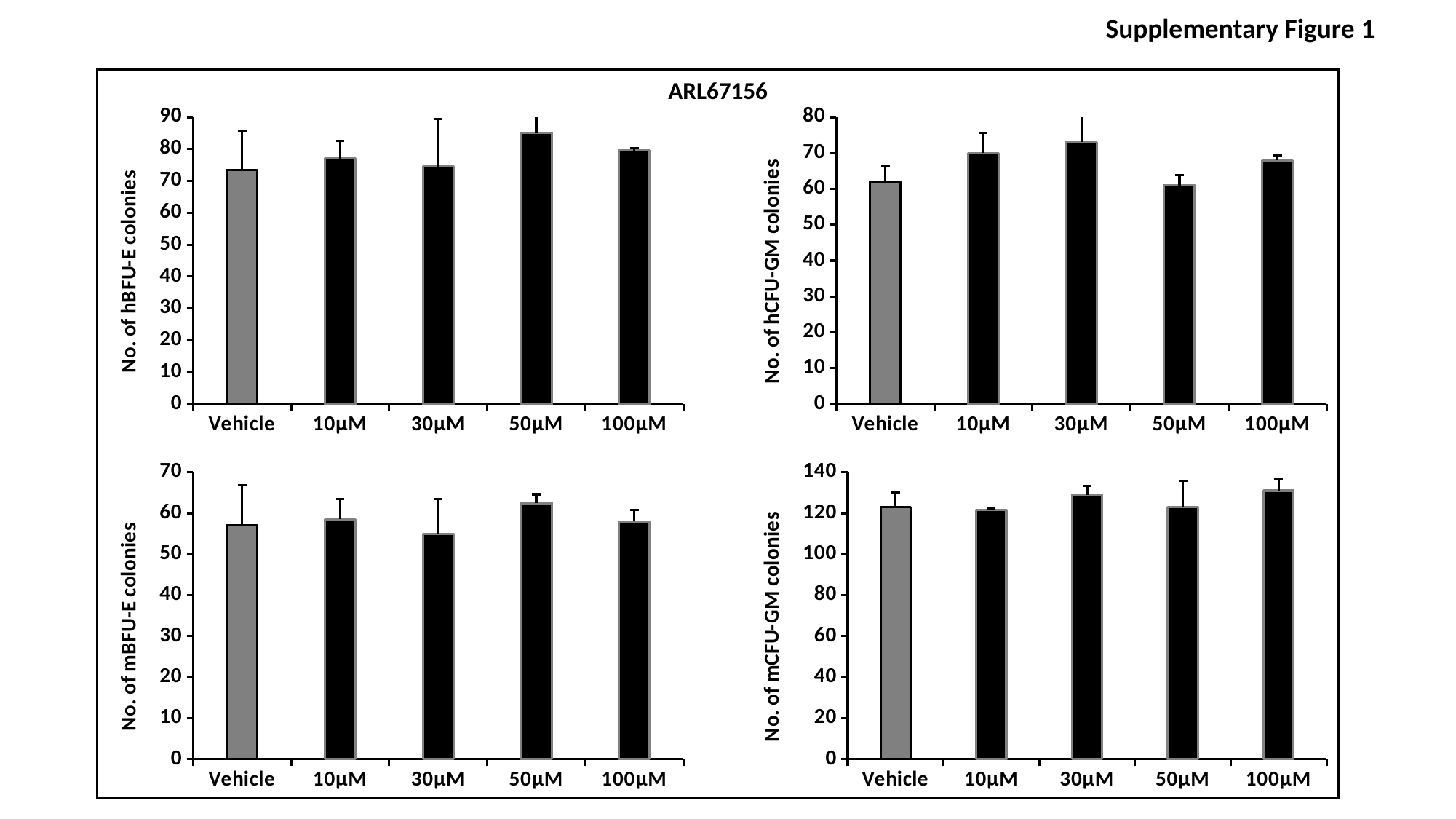

Supplementary Figure 1
ARL67156
### Chart
| Category | |
|---|---|
| Vehicle | 73.5 |
| 10µM | 77.0 |
| 30µM | 74.5 |
| 50µM | 85.0 |
| 100µM | 79.5 |
### Chart
| Category | |
|---|---|
| Vehicle | 62.0 |
| 10µM | 70.0 |
| 30µM | 73.0 |
| 50µM | 61.0 |
| 100µM | 68.0 |
### Chart
| Category | |
|---|---|
| Vehicle | 57.0 |
| 10µM | 58.5 |
| 30µM | 55.0 |
| 50µM | 62.5 |
| 100µM | 58.0 |
### Chart
| Category | |
|---|---|
| Vehicle | 123.0 |
| 10µM | 121.5 |
| 30µM | 129.0 |
| 50µM | 123.0 |
| 100µM | 131.0 |
