# Supplementary material for: The Inhibition of CD39 and CD73 Cell Surface Ectonucleotidases by Small Molecular Inhibitors Enhances the Mobilization of Bone Marrow Residing Stem Cells by Decreasing the Extracellular Level of Adenosine
Source: Stem Cell Rev Rep. 2019 Sep 13;15(6):892–9. doi: 10.1007/s12015-019-09918-y (PMC6925070; doi:10.1007/s12015-019-09918-y)
Supplement: Supplementary file 2 — Measurement of AMPCP toxicity. Murine BMMNC and human CD34+ cells were incubated for 1 h with different doses of CD73 inhibitor, than were resuspended in human methylcellulose base medium, supplemented with GM-CSF (25 ng/ml) and IL-3 (10 ng/ml) for determining the number of CFU-GM colonies and with thrombopoietin (TPO, 100 ng/ml) and IL-3 (10 ng/ml) for burst-forming unit-erythroid (BFU-E). Cultures were incubated for 7 and 14 days respectively (37 °C, 95% humidity, and 5% CO2), at which time they were scored under an inverted microscope for the number of colonies. Results from three independent experiments plated in duplicates are pooled together. (PPTX 53 kb) [file 12015_2019_9918_MOESM2_ESM.pptx]

## Slide 1
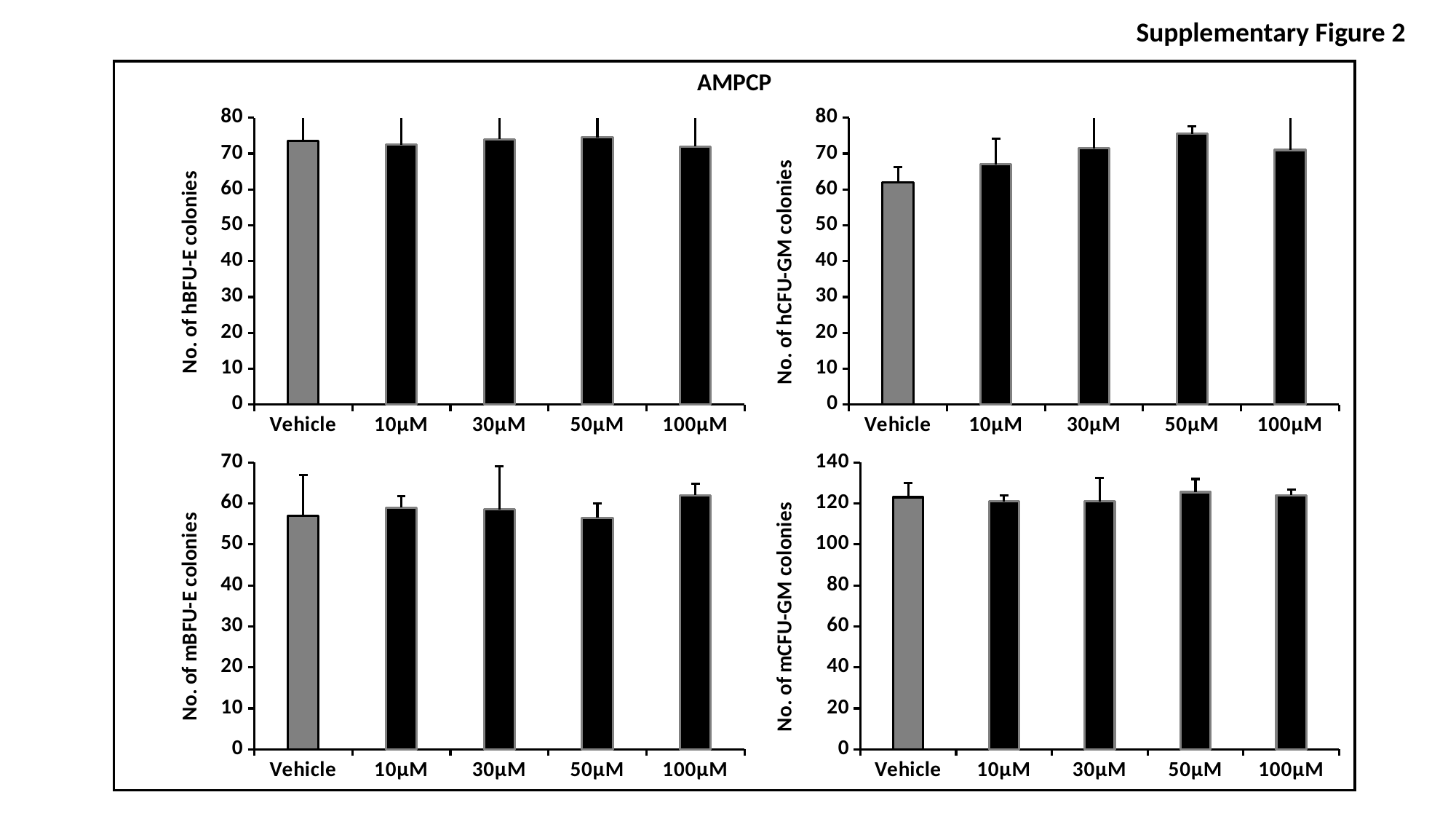

Supplementary Figure 2
AMPCP
### Chart
| Category | |
|---|---|
| Vehicle | 73.5 |
| 10µM | 72.5 |
| 30µM | 74.0 |
| 50µM | 74.5 |
| 100µM | 72.0 |
### Chart
| Category | |
|---|---|
| Vehicle | 62.0 |
| 10µM | 67.0 |
| 30µM | 71.5 |
| 50µM | 75.5 |
| 100µM | 71.0 |
### Chart
| Category | |
|---|---|
| Vehicle | 57.0 |
| 10µM | 59.0 |
| 30µM | 58.5 |
| 50µM | 56.5 |
| 100µM | 62.0 |
### Chart
| Category | |
|---|---|
| Vehicle | 123.0 |
| 10µM | 121.0 |
| 30µM | 121.0 |
| 50µM | 125.5 |
| 100µM | 124.0 |
